# Supplementary material for: “Take the treatment and be brave”: Care experiences of pregnant women with rifampicin-resistant tuberculosis
Source: PLoS One. 2020 Dec 21;15(12):e0242604. doi: 10.1371/journal.pone.0242604 (PMC7751874; doi:10.1371/journal.pone.0242604)
Supplement: S1 Appendix — (DOCX) [file pone.0242604.s001.docx]

Name: _____________________ Unique study no: ____________________

Surname: ___________________

**Previous pregnancies**

1. Have you ever been pregnant before/is this your first pregnancy?

……………………………………………………………………………………………………………………………………………………………………………………………………………………………………………………………………………………………………………………………………………………………….

1. How many children do you have?

……………………………………………………………………………………………………………………………………………………………………………….

1. How old are they?

……………………………………………………………………………………………………………………………………………………………………………….……………………………………………………………………………………………………………………………………………………………………………….

**Pregnancy and MDR-TB Rx**

1. When you were first diagnosed with MDR-TB did the doctor or any of the nurses explain the treatment to you? What did they say?

……………………………………………………………………………………………………………………………………………………………………………………………………………………………………………………………………………………………………………………………………………………………………………………………………………………………………………………………………………………………………………………………………………………………………………………………………………………………………………………………………………………………………………………………………………………………………………………………………………………………………………………………………………………

1. If the woman was **NOT** pregnant at the time of the MDR-TB diagnosis:

When you were first diagnosed with MDR-TB did the doctor or any of the nurses speak to you about contraceptives? What did they tell you?

…………………………………………………………………………………………………………………………………………………………………………………………………………………………………………………………………………………………………………………………………………………………………………………………………………………………………………………………………………………………………………………………………………………………………………………………………………………………………………………………………………………………………………

1. If the woman was pregnant at the time of the MDR-TB diagnosis:

Did the doctor or any of the nurses talk to you about MDR-TB Rx and it impact on your pregnancy? What did they say? **(Probe:** side effects of Rx on mother/baby)

………………………………………………………………………………………………………………………………………………………………………...

…………………………………………………………………………………………………………………………………………………………………………

………………………………………………………………………………………………………………………………………………………………………...

…………………………………………………………………………………………………………………………………………………………………………

1. How was your sense of well-being affected by the fact that you had to take MDR-TB Rx while pregnant?

………………………………………………………………………………………………………………………………………………………………………………………………………………………………………………………………………………………………………………………………………………………………………………………………………………………………………………………………………………………………………………………………

1. How was your sense of well-being in relation to anticipated child affected by the fact that you had to take MDR-TB Rx while pregnant? **(Probe: worries about future of the baby/how treatment would affect baby etc.)**

…………………………………………………………………………………………………………………………………………………………………………………………………………………………………………………………………………………………………………………………………………………………………………………………………………………………………………………………………………………………………………………………………………………………………………………………………………………………………………………………………………………………………………

1. If the woman has been pregnant before. Does this pregnancy feel the same to you as your previous

pregnancies? If different, please explain.

…………………………………………………………………………………………………………………………………………………………………………………………………………………………………………………………………………………………………………………………………………………………………………………………………………………………………………………………………………………………………………………………………………………………………………………………………………………………………………………………………………………………………………

1. Do you feel the baby moving in your stomach?

…………………………………………………………………………………………………………………………………………………………………………

1. If the mother has been pregnant before, ask her if this baby is as active as the last baby was.

……………………………………………………………………………………………………………………………………………………………………………………………………………………………………………………………………………………………………………………………………………………

1. Did you attend ANC whilst you were pregnant? If not, why not?

……………………………………………………………………………………………………………………………………………………………………………………………………………………………………………………………………………………………………………………………………………………

1. Where did you attend ANC? …………………………………………………………………………………………………….
2. When you were pregnant and taking MDR-TB treatment, for two or so hours after taking the treatment what did the baby do? (Probe: As active as during the rest of the day?)

…………………………………………………………………………………………………………………………………………………………………………………………………………………………………………………………………………………………………………………………………………………………………………………………………………………………………………………………………………………………………………………………………………………………………………………………………………………………………………………………………………………………………………

**Development of babies exposed to MDR-TB Rx in utero**

1. Did your baby reach his/her milestones **OR** did the baby sit, crawl, walk at the time other babies normally do? **(Probe: sitting before 12months, standing before 10months, walking a few steps before 12months, saying a few single words before 12months)** If not, please explain….

………………………………………………………………………………………………………………………………………………………………………………………………………………………………………………………………………………………………………………………………………………………………………………………………………………………………………………………………………………………………………………………………

1. **(if the baby was slow in reaching their milestone ask this question)** What kinds of emotions did you feel when you realised your baby was not reaching his/her milestones in time? **(Probe: Are you feeling anxious, helpless, cross with doctors, philosophical, bewildered)**

…………………………………………………………………………………………………………………………………………………………………………………………………………………………………………………………………………………………………………………………………………………………………………………………………………………………………………………………………………………………………………………………………………………………………………………………………………………………………………………………………………………………………………

**Treatment of pregnant women taking MDR-TB Rx**

1. If your very dear sister was pregnant and had to take MDR-TB medication, would you be concerned for your sister and her baby? If yes, why?

………………………………………………………………………………………………………………………………………………………………………………………………………………………………………………………………………………………………………………………………………………………………………………………………………………………………………………………………………………………………………………………………

1. What would you advise her to do?

………………………………………………………………………………………………………………………………………………………………………………………………………………………………………………………………………………………………………………………………………………………………………………………………………………………………………………………………………………………………………………………………

1. How do you think she should be treated? **(Note to Interviewer: More about how MDR-TB pregnancy should be managed)**

……………………………………………………………………………………………………………………………………………………………………………………………………………………………………………………………………………………………………………………………………………………

1. Is there anything else you would like to add that we have not covered in the above questions? Or any questions you have for me?

…………………………………………………………………………………………………………………………………………………………………………………………………………………………………………………………………………………………………………………………………………………………………………………………………………………………………………………………………………………………………………………………………………………………………………………………………………………………………………………………………………………………………………
