# Supplementary material for: “Take the treatment and be brave”: Care experiences of pregnant women with rifampicin-resistant tuberculosis
Source: PLoS One. 2020 Dec 21;15(12):e0242604. doi: 10.1371/journal.pone.0242604 (PMC7751874; doi:10.1371/journal.pone.0242604)
Supplement: Attachment — Submitted filename: Responses to Reviewers.docx [file pone.0242604.s003.docx]

**RESPONSES TO REVIEWERS**

PONE-D-20-13335R1
"Take the treatment and be brave": Care experiences of pregnant women with rifampicin-resistant tuberculosis
PLOS ONE

**Reviewer #1:**

Reviewer #1 notes that we have not made our data available. We have made our data available.

1. Abstract – revised satisfactorily. But please note the following:
a. The purpose of the COREQ guidelines is to check the quality of reporting of studies employing qualitative methodologies. The COREQ guidelines do not guide sampling strategy.
b. Please correct typo: “… 3) experience of the care they received for their pregnancy and their RR-TB; and 4) …”
**Response:** Thank you for raising these concerns. We have addressed these in the revised version of the manuscript which we have submitted.

2. Introduction – revised satisfactorily.

3. Methods and materials – revised satisfactorily. Suggested edits as follows:
a. Line 157: “This was a qualitative study generating data using open-ended interviews …”
b. Line 166: “The qualitative study was part of a larger cohort study of pregnant women …”
c. Line 169: “A convenience sample of 17 women was selected …”
d. Line 173: “The sample of 17 women participated in…”
**Response:** Thank you for these suggested corrections. We have revised the manuscript accordingly.

4. Results – revisions very acceptable!

5. Discussion – Revised satisfactorily. Suggested edits:
a. Generally I would recommend removing the definitive article ‘the’ when referring to ‘the women’. While you did indeed interview a specific group of respondents, and your findings are based on the data generated by these women, you are discussing a phenomenon, and your discussion should be transferrable to pregnant women in similar contexts; i.e. the discussion needs to be of the broader phenomenon of ‘care experiences of pregnant women with RR-TB’; and not of the women in this study (as you have done in the conclusion).
**Response:** Thank you for this suggestion. By removing ‘the’ together with some other minor edits the discussion is now more transferrable to pregnant women in similar contexts.

6. Limitations – Revised satisfactorily.
Reflexivity – statement that has been inserted is acceptable. However, I would recommend the authors read more extensively on the issues of reflexivity (epistemological, methodological, axiological, critical) and engage with the concept throughout the research process in future studies.

**Response:** Thank you for this suggestion. We will read more extensively on the issues of reflexivity for future studies.
